# Percutaneous Microwave Ablation Preserves Renal Function with Similar Long Term Oncologic Outcomes Compared to Surgery for Clinical T1 Renal Cell Carcinoma

**DOI:** 10.3390/cancers18020334

**Published:** 2026-01-21

**Authors:** Daniel F. Roadman, Daniel D. Shapiro, Arighno Das, Leslie W. Nelson, Paz Lotan, Michael C. Risk, Kyle A. Richards, Elizabeth L. Koehne, David F. Jarrard, Fred T. Lee, Glenn O. Allen, Edwarda Golden, Tim Ziemlewicz, James Louis Hinshaw, Edwin Jason Abel

**Affiliations:** 1 Department of Urology, University of Wisconsin School of Medicine and Public Health, Madison, WI 53792, USA; roadman@urology.wisc.edu (D.F.R.);; 2 Department of Radiology, University of Wisconsin School of Medicine and Public Health, Madison, WI 53792, USA; lnelson8@uwhealth.org (L.W.N.); edesouza@wisc.edu (E.G.);

**Keywords:** renal cell carcinoma, microwave ablation, partial nephrectomy, radical nephrectomy, small renal masses, oncologic outcomes

## Abstract

**Background/Objectives:** Percutaneous microwave (MW) ablation is a nephron sparing treatment for localized renal cell carcinoma (RCC). We compared perioperative, renal functional, and oncologic outcomes for clinical stage 1 RCC treated with MW ablation, PN, or RN. **Methods:** Adults with clinical T1 kidney masses treated with MW ablation, PN, or RN from 2001–2025 were identified. Outcomes included: 90-day overall and major complication rate, 30-day readmission rate, length of hospital stay (LOS), change in renal function, local recurrence-free survival (LRFS), metastasis-free survival (MFS), and cancer-specific survival (CSS). Univariable and multivariable analyses evaluated outcomes adjusted for confounders. **Results:** A total of 2201 patients with renal masses ≤ 7 cm and no evidence of locally advanced or metastatic disease were treated with MW ablation (708), PN (729), or RN (764). MW ablation patients were older and more comorbid compared to both PN/RN, whereas RN patients had larger, higher-grade tumors. Ninety-day overall complications were lowest after MW ablation (8.9% vs. 20.3% PN, *p* < 0.001 and 8.9% vs. 19.9% RN, *p* < 0.001). LOS was shortest after MW ablation (median 1 day vs. 3 days PN/RN, *p* < 0.001 for each). Six-month eGFR decline was similar after MW ablation and PN (−5.2% and −4.7%, *p* = 0.84) but greater after RN (−32.9%, *p* < 0.001). Local recurrences were more common with MW ablation, with five-year LRFS 96.4% versus 99.7% for PN (*p* < 0.001). Five-year MFS (99.5% vs. 99.7%, *p* = 0.24) and CSS (99.3% vs. 99.7%, *p* = 0.71) did not differ between MW ablation and PN. **Conclusions:** Percutaneous MW ablation has comparable metastasis free and cancer specific survival with lower perioperative morbidity and comparable renal preservation to PN, despite worse baseline comorbidity and renal function. These findings support MW ablation as an effective nephron-sparing option for appropriately selected patients with clinical T1 RCC when performed at an experienced center.

## Simple Summary

Microwave ablation is a minimally invasive, nephron-sparing treatment increasingly used for localized renal cell carcinoma, particularly in older or medically complex patients. However, long-term comparative data evaluating oncologic outcomes relative to surgery remain limited. In this study, we compared perioperative, renal functional, and oncologic outcomes among 2201 patients with clinical T1a and T1b renal masses treated with microwave ablation, partial nephrectomy, or radical nephrectomy. Patients treated with microwave ablation experienced significantly lower perioperative morbidity, shorter hospital stays, and renal functional preservation comparable to partial nephrectomy, despite greater baseline comorbidity. While local recurrence occurred more frequently following microwave ablation, metastasis-free and cancer-specific survival were similar to partial nephrectomy after adjustment for confounders. These findings support microwave ablation as an effective nephron-sparing treatment option for appropriately selected patients with clinical T1 renal cell carcinoma.

## 1. Introduction

Increased cross-sectional imaging has led to increased incidence and treatment of incidentally detected clinical T1 (cT1) renal masses, which now account for more than half of newly diagnosed renal cell carcinoma (RCC) cases, although kidney cancer mortality has improved only slightly [1,2,3,4]. This realization has intensified efforts to balance durable oncologic control with renal functional preservation, perioperative morbidity, and competing comorbidity risks [5]. For cT1a tumors (≤4 cm), treatment options include active surveillance (AS), thermal ablation (TA), and surgery. For cT1b tumors (4.1–7 cm), partial nephrectomy (PN) or radical nephrectomy (RN) are standard approaches, although microwave (MW) ablation has theoretical technical advantages that may enable ablation of larger tumors in selected medically complex patients or those unable to undergo surgery [6].

Interpretation of comparative studies is complicated by baseline comorbidity differences, as well as differences in tumor histology and anatomic complexity across treatment groups, all of which influence treatment selection [2,7]. Especially in older, comorbid patients, TA of renal tumors (≤3 cm) provides treatment with minimal risk of procedural complications, and discussion of TA is endorsed in contemporary small renal mass (SRM) guidelines [6,8,9,10,11,12,13,14]. Radiofrequency ablation and cryoablation are established modalities, but MW ablation offers potential advantages, particularly over radiofrequency ablation, including more uniform tissue heating, larger predictable ablation zones, and reduced heat-sink effect [6,15]. Early experiences demonstrated promising local control and low complication rates, and subsequent analyses showed renal functional preservation comparable to PN when baseline eGFR and comorbidity burden are considered [16,17,18]. However, early population-based comparisons suggested inferior survival with TA relative to PN [19]. These comparisons were confounded by lack of tumor complexity metrics, comorbidity data, and renal function information, introducing substantial confounding variables by indication, and limiting external validity. Indeed, subsequent analyses suggested that apparent survival advantages of PN in Surveillance, Epidemiology, and End Results program population level data largely reflected confounded treatment effects rather than intrinsic oncologic superiority [20].

Contemporary institutional series including a small, randomized trial, propensity-matched cohorts and meta-analytic series now suggest comparable cancer-specific and metastasis-free survival between TA and PN, with slightly higher local recurrence risk after TA but reduced procedural morbidity [21,22,23,24,25]. Importantly, most comparative TA literature has combined radiofrequency, cryoablation, and MW ablation cohorts, limiting the ability to evaluate modality-specific oncologic performance [11,23,26,27]. Additionally, most TA cohorts have been limited to T1a disease, while carefully selected T1b tumors are now increasingly managed with MW ablation in modern practice, however, long-term comparative data for T1b masses remains limited [6,13,27,28,29]. To address these gaps, we present to date the largest series evaluating perioperative, renal functional, and oncologic outcomes among patients with clinical T1a and T1b renal masses treated with MW ablation, PN, or RN at a high-volume academic center.

## 2. Materials and Methods

A prospectively maintained institutional database was queried to identify adults (age ≥ 18) with solid or predominantly solid renal masses ≤ 7cm and with primary treatment being either MW ablation, PN, or RN from 2001–2025 after institutional review board approval. Patients were excluded if there was a known renal vein (n = 34) or inferior vena cava thrombus (n = 32), or metastatic disease (n = 43) at the time of treatment. Patients with multiple treated tumors (n = 312, including multifocal, bilateral tumors, hereditary RCC, and tumors in solitary kidneys) were excluded from renal functional analyses. Among the remaining patients, those without RCC were excluded from oncologic analyses (n = 229, including non-RCC malignancy, benign/indeterminate tumors, pathology unknown) (Figure S1). Complete patient data was collected and analyzed. Patients were divided into cohorts based on treatment modality (MW ablation, PN, or RN). MW ablation procedures were performed percutaneously in a CT suite with ultrasound (US) and/or CT fluoroscopy for image-guidance. A 2.45 GHz, gas-cooled MWA device (Certus 140, NeuWave Medical, Madison, WI, USA) with 15- or 17-gauge antennas (PR or LK, NeuWave Medical) was used for all cases. Baseline clinical, demographic, and pathologic differences across treatment groups were analyzed. Continuous variables were summarized using medians and interquartile ranges and compared using Kruskal–Wallis tests for overall differences, with pairwise Wilcoxon rank-sum tests for MW ablation vs. PN, MW ablation vs. RN, and PN vs. RN. Categorical variables were compared using Fisher exact tests or chi-square tests for both overall and pairwise comparisons.

Perioperative outcomes included 90-day postoperative complications classified using the Clavien-Dindo method [30], 30-day hospital readmission, length of hospital stay (LOS), and percentage change in estimated glomerular filtration rate [31] (eGFR) at initial postoperative assessment and 6-months. Logistic and linear regression models were used to evaluate for associations between treatment modalities and perioperative outcomes adjusting for other known prognostic variables.

Oncologic survival outcomes were evaluated for patients who had pathologic diagnosis of RCC using Kaplan Meier analysis. Surveillance imaging after MW ablation was obtained every 6-months for 2 years and annually thereafter consisting of contrast-enhanced abdominal CT or magnetic resonance imaging and chest imaging per institutional practice. Surveillance imaging after PN or RN was performed identically with adjustment as indicated based on guideline stage appropriate recommendations [14,32]. Patients were evaluated for local recurrence-free survival (LRFS), metastasis-free survival (MFS), and cancer-specific survival (CSS). Survival distributions stratified by treatment modality were compared using the log-rank test. Cox proportional hazards models were used to evaluate for associations between treatment modality and survival outcomes adjusting for other confounding variables prespecified to reflect key clinical domains at the time of treatment decision-making including patient health (age and CCI), tumor characteristics (tumor size and histologic features), and baseline renal reserve (pre-treatment eGFR). MW ablation was introduced at our institution in 2011; therefore, a sensitivity analysis restricted to patients treated from 2011–2025 was performed to ensure that earlier-era surgical cases were not driving the observed oncologic associations. As a sensitivity analysis, 1:1 propensity score matching was also performed within clinical T stage (cT1a and cT1b) using restrictions of ±7 years for age, ±1 point for CCI, ±15 mL/min/1.73 m^2^ for baseline eGFR, ±2 cm for radiographic tumor size, and ±2 points for nephrometry score. For comparisons involving RN, restrictions for CCI were expanded to ±2 points when necessary to achieve adequate matching due to limited matching.

Local recurrence after MW ablation was defined as new or enlarging contrast-enhancing nodularity within the ablation zone after an initially non-enhancing post-ablation cavity, and primary technical efficacy was defined as complete ablation without residual enhancement on initial post-procedural imaging, consistent with consensus time-to-event definitions for image-guided tumor ablation [33]. Local recurrence after PN was defined as a new enhancing mass within the ipsilateral kidney. Local recurrence after RN was defined as enhancing soft tissue in the nephrectomy bed. An expert abdominal radiologist (LN) who was not part of the ablation treatment team independently reviewed all imaging for radiographic evidence of tumor recurrences for this study. Initial treatment modality (MW ablation, PN, or RN) was determined through shared decision making in clinic between Urology faculty and the patient considering patient age, comorbidities, tumor size, location and histology, and proximity of non-target anatomy on an individual basis. All patients treated with MW ablation were discussed in a multidisciplinary conference prior to ablation ensuring technical feasibility. Data analyses were performed using STATA version 19.0 (Statacorp, College Station, TX, USA). For all statistical tests, a *p* value of less than 0.05 was considered statistically significant.

## 3. Results

A total of 2201 adult patients with renal masses ≤ 7 cm and no evidence of locally advanced or metastatic disease were treated with MW ablation (708), PN (729), or RN (764). Patient and disease characteristics are shown in Table 1. MW ablation patients were older (median age 67.5 years vs. 55.0 PN and 61.5 RN; *p* < 0.001 for all) and had greater comorbidity burden (Charlson Comorbidity Index excluding age (CCI): median 2 vs. 0 PN and 1 RN; *p* < 0.001 for all). Tumors in solitary kidneys and bilateral tumors were more common in the MW ablation cohort compared to the surgical cohorts (solitary kidney 9.3% vs. 1.9% PN (*p* < 0.001) and 0.8% RN (*p* < 0.001); bilateral tumors 10.2% vs. 4.9% PN (*p* < 0.001) and 5.4% RN (*p* < 0.001). MW ablation patients had shorter follow-up than surgical cohorts (55.2 months [IQR 31.2–91.2], PN 86.4 months [IQR 36.0–132.0], RN 75.6 months [IQR 31.2–144.0]; *p* < 0.001). Tumors treated with PN or MW ablation had similar tumor diameters and complexity measured by nephrometry score. Tumors treated with RN were larger compared to MW ablation or PN (median diameter 4.5 cm RN vs. 2.7 cm MW ablation vs. 2.6 cm PN) and more complex (median nephrometry score 9 [IQR 7–10] for RN vs. 6 [5,6,7,8] MW ablation and PN; *p* < 0.001). MW ablation patients exhibited several baseline clinical differences, including older age (*p* < 0.001), higher rates of diabetes (*p* < 0.001) and hypertension (*p* < 0.001), higher BMI (*p* = 0.02), and an increased prevalence of bilateral tumors (*p* < 0.001) and solitary kidney tumors (*p* < 0.001).

Supplemental Table S1 demonstrates the patient and disease characteristics for patients with a pathological diagnosis of RCC (Table S1). Similar to the overall cohort, MW ablation patients were older, had higher comorbidity scores, a slight male predominance, and were more likely to have diabetes, hypertension, and bilateral tumors. With regard to RCC histologic subtype, MW ablation patients were more likely to have papillary RCC (19.6% MW ablation vs. 14.6% PN vs. 11.2% RN). Patients treated with MW ablation also had fewer nuclear grade 3 and 4 tumors (6.7% MW ablation vs. 24.1% PN vs. 36.1% RN).

Perioperative outcomes differed among modalities and are shown in Table 2. Median length of stay (LOS) after the procedure was 1 day for MW ablation and 3 days for both PN and RN (*p* < 0.001). On multivariable logistic regression, MW ablation was strongly associated with lower risk of prolonged hospitalization > 3 days. In the multivariable model, MW ablation remained independently associated with lower odds of prolonged LOS (OR 0.03; 95% CI 0.02–0.06; *p* < 0.001), whereas RN again showed no statistically significant difference relative to PN (*p* = 0.28). Age, tumor size, comorbidity, and open surgery all remained significant independent predictors of prolonged LOS (each *p* < 0.001) (Table 3).

90-day overall complication rates were lowest after MW ablation (8.9%) compared with PN (20.3%, *p* < 0.001) and RN (19.9%, *p* < 0.001). Major (Clavien ≥ 3) complications were uncommon and no difference in rates was identified among modalities (4.5% MW ablation compared to 4.1% PN (*p* = 0.80) and 4.8% RN (*p* = 0.81)). Most commonly reported major complications included bleeding requiring embolization, collecting system injuries requiring ureteral stent or nephrostomy tube placement, cardiac or respiratory events, and severe infections requiring intensive care unit management. In the multivariable model adjusting for age, tumor size, comorbidity index, and surgical approach, choice of MW ablation vs. PN (*p* = 0.24) was not associated with higher risk of major complications. Within this model, age (OR 1.03; 95% CI 1.01–1.06; *p* = 0.02), tumor size (OR 1.33; 95% CI 1.08–1.63; *p* = 0.01), and CCI (OR 1.29; 95% CI 1.13–1.46; *p* < 0.001) were independent predictors of major complications.

Thirty-day readmission rates were low, and no differences were identified among treatment types (4.2% MW ablation, 4.3% PN, 5.4% RN; *p* = 0.50 for all) (Table 2). In multivariable analysis, CCI remained the only independent predictor of readmission (OR 1.26; 95% CI 1.14–1.39; *p* < 0.001).

Preoperative baseline renal function was lowest in patients treated with MW ablation (median eGFR 69.0 mL/min/1.73 m^2^ vs. 79.6 mL/min/1.73 m^2^ PN (*p* < 0.001) and 71.9 mL/min/1.73 m^2^ RN (*p* < 0.001)). Immediate postoperative eGFR decline was smallest after MW ablation and greatest after RN (median percent change −5.6% MW ablation, −11.2% PN, −34.8% RN; *p* < 0.001 for all). At 6-months, renal preservation remained similar between MW ablation and PN (median −5.2% vs. −4.7%; *p* = 0.84) but substantially worse after RN (−32.9%; *p* < 0.001 vs. each of MW ablation and PN). In the multivariable model adjusting for baseline eGFR, hypertension, and diabetes, RN was independently associated with greater eGFR decline at 6-months (β −16.06 mL/min/1.73 m^2^ (95% CI −22.86–−9.26); *p* < 0.001) (Table 4). At 6-months, patients treated with MW ablation had no significant difference in eGFR decline from PN (β –2.68 mL/min/1.73 m^2^; (95% CI −8.97–3.62); *p* = 0.41). Baseline eGFR was a strong predictor of postoperative renal decline (β –0.44 mL/min/1.73 m^2^; (95%CI −0.55–−0.34); *p* < 0.001), whereas neither hypertension nor diabetes remained independently associated after adjustment.

**Figure 1:**
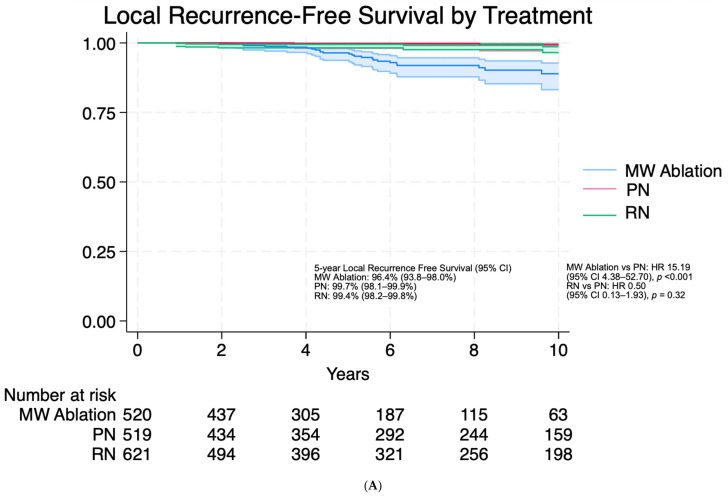

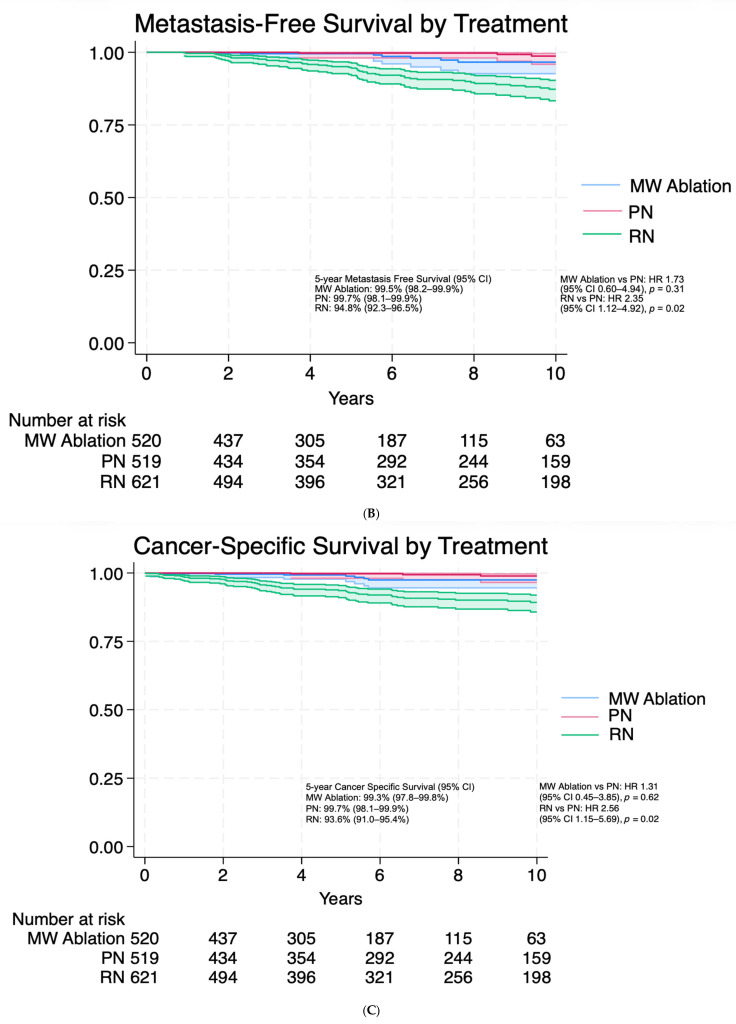
Unadjusted Kaplan–Meier survival curves for localized renal cell carcinoma by treatment modality among patients treated with Microwave (MW) Ablation, Partial Nephrectomy (PN), or Radical Nephrectomy (RN) demonstrating (**A**) local recurrence–free survival, (**B**) metastasis-free survival, (**C**) cancer-specific survival.

Oncologic outcomes were assessed for patients with pathologically diagnosed RCC tumors with no prior treatment and without hereditary/multifocal/bilateral tumors and no prior RCC history. Median follow-up was 67 [33–120] months following treatment. Unadjusted Kaplan-Meier estimates for LRFS, MFS, and CSS are shown in Figure 1. Size-stratified analyses are provided in Tables S2–S4. After multivariable Cox proportional hazards analysis adjusting for treatment approach, age, tumor size, histologic subtype, and Fuhrman grade, long-term MFS (*p* = 0.31) and CSS (*p* = 0.62) were similar across MW ablation and PN groups (Table 5). For MFS, independent predictors of metastases included RN (HR 2.35, 95% CI 1.12–4.92; *p* = 0.02), age (HR 1.02, 95% CI 1.00–1.04; *p* = 0.04), tumor size per cm (HR 1.55, 95% CI 1.3201.84; *p* < 0.001), and nuclear grade (HR 3.75, 95% CI 2.33 6.03; *p* < 0.001). Similarly for CSS, independent predictors of cancer specific death included RN (HR 2.56, 95% CI 1.15–5.69; *p* = 0.02), tumor size per cm (HR 1.39, 95% CI 1.17–1.67; *p* < 0.001), and nuclear grade (HR 2.97, 95% CI 1.79–4.93; *p* < 0.001). In a sensitivity analysis restricted to patients treated from 2011–2025, corresponding to the period during which MW ablation was performed at our institution, associations for LRFS, MFS, and CSS were consistent with the primary analysis (Table S5). We additionally performed 1:1 propensity score matching within clinical T stage (Table S6); HR estimates remained consistent, although sparse events limited estimability, particularly within cT1b (Table S7).

MW ablation was associated with higher local recurrence risk compared with PN (*p* < 0.001). Management of local recurrences for MW ablation patients included repeat TA (n = 14), AS (n = 8, median 28.0 months on AS), surgery (n = 4), stereotactic radiation (n = 2), and systemic therapy (n = 1) for metastatic progression combined with local recurrence. Of the 14 patients treated with salvage TA for MW ablation failure, repeat TA was associated with durable secondary local control, with a secondary efficacy of 100% at a median follow-up of 44.5 months. Salvage management of surgical treatment (PN/RN) local recurrence included TA (n = 3), completion nephrectomy (n = 3), active surveillance (n = 1), stereotactic radiation (n = 1), and systemic therapy for early metastatic progression (n = 1).

**Table 1:** Baseline Clinical, Demographic, and Tumor Characteristics of the Perioperative Outcomes Cohort by Treatment Modality (n = 2201).

	MW Ablation N = 708	PN N = 729	RN N = 764	*p* Value MW Ablation vs. PN	*p* Value MW Ablation vs. RN	*p* Value PN vs. RN	*p* Value Overall
Median Age, y, (IQR)	67.5 [61.6, 73.9]	55.0 [45.4, 62.9]	61.5 [53.0, 69.1]	<0.001	<0.001	<0.001	<0.001
Gender: Male n (%)	488 (68.9%)	426 (58.4%)	490 (64.1%)	<0.001	0.06	0.03	<0.001
Race n (%)							
White	650 (91.8%)	679 (93.1%)	703 (92.0%)	0.48	0.70	0.71	0.75
African American	32 (4.5%)	24 (3.3%)	29 (3.8%)				
Other	26 (3.7%)	26 (3.6%)	32 (4.2%)				
Median BMI, (IQR)	31.2 [26.8, 36.6]	30.5 [26.5, 35.7]	30.2 [26.6, 35.1]	0.09	0.01	0.29	0.02
Smoking History n (%)	310 (43.8%)	349 (47.9%)	420 (55.0%)	0.13	<0.001	0.01	<0.001
Diabetes History n (%)	218 (30.8%)	127 (17.4%)	187 (24.5%)	<0.001	0.007	<0.001	<0.001
Hypertension n (%)	501 (70.8%)	337 (46.2%)	398 (52.1%)	<0.001	<0.001	0.03	<0.001
Solitary Kidney n (%)	66 (9.3%)	14 (1.9%)	6 (0.8%)	<0.001	<0.001	0.07	<0.001
Bilateral Tumor n (%)	72 (10.2%)	36 (4.9%)	41 (5.4%)	<0.001	<0.001	0.73	<0.001
Multifocal Tumor n (%)	45 (6.4%)	36 (4.9%)	43 (5.6%)	0.26	0.58	0.57	0.51
Charlson comorbidity index, excluding age, (IQR)	2 [1, 4]	0 [0, 1]	1 [0, 2]	<0.001	<0.001	<0.001	<0.001
Median Radiographic tumor diameter, cm, (IQR)	2.7 [2, 3.5]	2.6 [2, 3.7]	4.5 [3.4, 5.8]	0.863	<0.001	<0.001	<0.001
Median Nephrometry score (IQR)	6 [5, 8]	6 [5, 8]	9 [7, 10]	0.25	<0.001	<0.001	<0.001
Tumor histologic subtype n (%)							
Clear cell RCC	447 (63.1%)	449 (61.6%)	558 (73.0%)	<0.001	<0.001	<0.001	<0.001
Papillary RCC	131 (18.5%)	87 (11.9%)	78 (10.2%)				
Chromophobe RCC	32 (4.5%)	43 (5.9%)	28 (3.7%)				
RCC Other *	58 (8.2%)	19 (2.6%)	32 (4.2%)				
Malignant Other ^†^	1 (0.1%)	7 (1.0%)	8 (1.0%)				
Benign Other °	22 (3.1%)	124 (17.0%)	60 (7.9%)				
Pathology Unknown	17 (2.4%)	0 (0.0%)	0 (0.0%)				
Surgical approach n (%)							
Open	0 (0.0%)	330 (45.3%)	186 (24.3%)	<0.001	<0.001	<0.001	<0.001
Laparoscopic/Robotic	0 (0.0%)	399 (54.7%)	578 (75.7%)				
Percutaneous	708 (100.0%)	0 (0.0%)	0 (0.0%)				

MW = Microwave, PN = Partial Nephrectomy, RN = Radical Nephrectomy. * RCC Other Included: Clear Cell Papillary RCC (n = 68), RCC Unspecified (n = 11), RCC Unclassified (n = 19), Collecting Duct Carcinoma (n = 2), Renal Medullary Carcinoma (n = 1), Translocation RCC (n = 5), Succinate dehydrogenase deficient RCC (n = 3). † Malignant Other included: Squamous Cell Carcinoma (n = 1), Sarcoma (n = 3), Adenocarcinoma (n = 1) newly diagnosed metastatic cancer from unknown site (n = 9), Large Cell Carcinoma (n = 1). ° Benign Other included: Oncocytoma (n = 96), Angiomyolipoma (n = 55), eosinophilic vacuolated tumor (n = 2), pheochromocytoma (n = 1), myxoid pseudotumor (n = 1), mixed epithelial and stromal tumor (n = 2), juxtaglomerular tumor (n = 2), Xanthogranulomatous pyelonephritis (n = 3), benign cyst (n = 43), anastomosing hemangioma (n = 1), adrenal adenoma (n = 1).

**Table 2:** Perioperative, Readmission, Complications, and Renal Function Outcomes by Treatment Modality.

	MW Ablation N = 708	PN N = 729	RN N = 764	*p* Value MW Ablation vs. PN	*p* Value MW Ablation vs. RN	*p* Value PN vs. RN	*p* Value Overall
Median Hospital Length of Stay (days) (IQR)	1 [1, 1]	3 [2, 4]	3 [2, 4]	<0.001	<0.001	0.001	<0.001
Readmission within 30-days n (%)	30 (4.2%)	31 (4.3%)	41 (5.4%)	1.0	0.33	0.34	0.50
Complication within 90-days n (%)	63 (8.9%)	148 (20.3%)	152 (19.9%)	<0.001	<0.001	0.85	<0.001
Clavien-Dindo Grade 1–2	31 (4.4%)	118 (16.2%)	115 (15.0%)	<0.001	<0.001	0.41	<0.001
Clavien-Dindo Grade 3–5	32 (4.5%)	30 (4.1%)	37 (4.8%)	0.80	0.81	0.53	0.80
Preoperatively eGFR	69.0 [52.8, 85.2]	79.6 [67.6, 93.7]	71.9 [56.2, 86.1]	<0.001	0.17	<0.001	<0.001
Immediate postoperative percent change	−5.6 [−15.4, 3.4]	−11.2 [−26.5, 0]	−34.8 [−42.0, −22.7]	<0.001	<0.001	<0.001	<0.001
6-months postoperative percent change	−5.2 [−15.4, 4.4]	−4.7 [−14.5, 4.5]	−32.9 [−41.8, −22.7]	0.84	<0.001	<0.001	<0.001

MW = Microwave, PN = Partial Nephrectomy, RN = Radical Nephrectomy.

**Table 3:** Univariable and Multivariable Logistic Regression Models for Perioperative Outcomes.

Predictors	Univariable		Multivariable	
	OR (95% CI)	*p*-Value	OR (95% CI)	*p*-Value
Major Complication (Clavien ≥ 3)				
MW Ablation vs. PN	1.03 (0.49–2.18)	0.94	0.55 (0.20–1.5)	0.24
RN vs. PN	1.87 (0.97–3.59)	0.06	0.96 (0.42–2.17)	0.92
Age (per year)	1.04 (1.02–1.07)	<0.001	1.03 (1.01–1.06)	0.02
Tumor size (per cm)	1.36 (1.16–1.61)	<0.001	1.33 (1.08–1.63)	0.01
Charlson comorbidity index (no age)	1.29 (1.15–1.44)	<0.001	1.29 (1.13–1.46)	<0.001
Open vs. minimally invasive/percutaneous	1.90 (1.09–3.32)	0.02	1.49 (0.73–3.01)	0.27
30-Day Readmission				
MW Ablation vs. PN	1.00 (0.60–1.66)	0.99	0.90 (0.45–1.82)	0.78
RN vs. PN	1.28 (0.79–2.06)	0.32	1.31 (0.71–2.41)	0.39
Age (per year)	0.99 (0.98–1.01)	0.33	0.98 (0.96–1.00)	0.06
Tumor size (per cm)	1.01 (0.89–1.15)	0.91	0.96 (0.82–1.13)	0.63
Charlson comorbidity index (no age)	1.21 (1.10–1.32)	<0.001	1.26 (1.14–1.39)	<0.001
Open vs. minimally invasive/percutaneous	1.45 (0.94–2.24)	0.09	1.09 (0.63–1.90)	0.75
Length of Stay > 3 Days				
MW Ablation vs. PN	0.03 (0.02–0.05)	<0.001	0.03 (0.02–0.06)	<0.001
RN vs. PN	1.21 (0.98–1.49)	0.07	1.18 (0.87–1.61)	0.28
Age (per year)	0.99 (0.99–1.00)	0.11	1.02 (1.01–1.03)	<0.001
Tumor size (per cm)	1.39 (1.31–1.48)	<0.001	1.21 (1.11–1.33)	<0.001
Charlson comorbidity index (no age)	0.96 (0.92–1.02)	0.16	1.20 (1.11–1.29)	<0.001
Open vs. minimally invasive/percutaneous	11.41 (9.10–14.32)	<0.001	6.14 (4.66–8.09)	<0.001

MW = Microwave, PN = Partial Nephrectomy, RN = Radical Nephrectomy.

**Table 4:** Univariable and Multivariable Linear Regression for percentage change in 6-month eGFR.

	Univariable			Multivariable		
	β	95% CI	*p*-Value	β	95% CI	*p*-Value
RN vs. PN	−20.35	−26.50–−14.20	<0.001	−16.06	−22.86–−9.26	<0.001
MW Ablation vs. PN	1.85	−4.46–8.16	0.57	−2.68	−8.97–3.62	0.41
Tumor size (cm)	−7.00	−8.64–−5.37	<0.001	−4.91	−6.78–−3.05	<0.001
Hypertension	3.20	−2.01–8.42	0.23	−1.75	−6.92–3.43	0.51
Diabetes	6.26	0.41–12.10	0.04	5.11	−0.60–10.83	0.08
Baseline eGFR (mL/min/1.73 m^2^)	−0.41	−0.51–−0.31	<0.001	−0.44	−0.55–−0.34	<0.001

MW = Microwave, PN = Partial Nephrectomy, RN = Radical Nephrectomy.

**Table 5:** Multivariable Cox Proportional Hazards Models Adjusted for Treatment, Age, Tumor Size, Histologic Subtype, and Fuhrman Grade.

	Comparison	Multivariable	
		HR (95% CI)	*p*-Value
Local Recurrence Free Survival			
	MW Ablation vs. PN	15.19 (4.38–52.70)	<0.001
	RN vs. PN	0.50 (0.13–1.93)	0.32
	Age (years)	1.04 (1.00–1.08)	0.04
	Tumor size (cm)	1.31 (1.04–1.66)	0.02
	Histology: Clear Cell Referent:		
	Papillary RCC	0.66 (0.25–1.73)	0.40
	Chromophobe/other RCC	0.39 (0.09–1.66)	0.20
	High Nuclear Grade (3–4) vs. Low (1–2)	4.25 (1.87–9.68)	<0.001
Metastasis Free Survival			
	MW Ablation vs. PN	1.73 (0.60–4.94)	0.31
	RN vs. PN	2.35 (1.12–4.92)	0.02
	Age (years)	1.02 (1.00–1.04)	0.04
	Tumor size (cm)	1.55 (1.32–1.84)	<0.001
	Histology: Clear Cell Referent:		
	Papillary RCC	0.79 (0.36–1.75)	0.56
	Chromophobe/other RCC	0.88 (0.35–2.19)	0.78
	High Nuclear Grade (3–4) vs. Low (1–2)	3.75 (2.33–6.03)	<0.001
Cancer Specific Survival			
	MW Ablation vs. PN	1.31 (0.45–3.85)	0.62
	RN vs. PN	2.56 (1.15–5.69)	0.02
	Age (years)	1.02 (1.00–1.05)	0.05
	Tumor size (cm)	1.39 (1.17–1.67)	<0.001
	Histology: Clear Cell Referent:		
	Papillary RCC	0.62 (0.25–1.57)	0.31
	Chromophobe/other RCC	1.16 (0.50–2.71)	0.73
	High Nuclear Grade (3–4) vs. Low (1–2)	2.97 (1.79–4.93)	<0.001

MW = Microwave, PN = Partial Nephrectomy, RN = Radical Nephrectomy.

## 4. Discussion

In this large comparative treatment cohort study of patients with clinical T1 RCC, MW ablation demonstrated similar metastasis free and cancer specific survival with lower risk for perioperative morbidity and similar renal functional preservation. This project differs from most prior renal mass ablation studies because it includes larger T1b tumors, utilized an independent radiology review for recurrences, and compared directly to common surgical treatments (PN and RN) [6,28]. Local recurrences were more common in patients treated with MW ablation, but consistent with prior studies were frequently salvaged with a second ablation [34]. Despite older patients and more comorbidities in the MW ablation cohort, hospitalization was shorter after MW ablation compared to surgery and overall complication rates were lower. As observed in other studies, local recurrence was more common in MW ablation but compares favorably to other TA modalities with five-year LRFS of 96.4%. As such, these data demonstrate that MW ablation offers an alternative to surgery with low complication rates, comparable 5-year CSS outcomes and excellent renal preservation for selected patients.

Perioperative safety and short-term recovery favored MW ablation. In this cohort, 90-day overall complication rates were lowest after MW ablation compared with PN and RN, whereas major (Clavien ≥ 3) complications did not differ across modalities but occurred in less than 5% of all treatment modalities. Adjusted models demonstrated that age, tumor size, and level of comorbidities but not choice of treatment were independently predictive of major complications. These findings align with meta-analytic data in which ablation is associated with significantly lower overall complication rates than PN (13% vs. 17.6%) [26]. Similarly, 30-day readmission rates were low among treatment groups (4.2% MW ablation, 4.3% PN, 5.4% RN), and comorbidity burden, rather than treatment type, was the only independent predictor of readmission. Median hospital LOS was 1 day after MW ablation versus 3 days after both PN and RN, and MW ablation remained independently associated with a markedly lower odds of prolonged hospitalization after adjustment for age, tumor size, comorbidity, and surgical approach. This is likewise consistent with recent reviews of MW ablation that report low, predominantly minor complication rates and hospital stays that are 1.8–3.6 days shorter than surgery, supporting our observation that MW ablation delivers durable treatment with low serious-complication risk and rapid recovery in appropriately selected patients [35]. Collectively, these data demonstrate safety and favorable perioperative outcomes for MW ablation of renal masses.

Renal functional preservation is critical when feasible and contributes significantly to patients mortality. In this study, a smaller immediate postoperative decline in eGFR was observed after MW ablation; this difference likely reflects a combination of peri-procedural and selection-related factors because MW ablation does not require temporary kidney ischemia utilized in most PN. At 6-months following treatment, MW ablation and PN had similar rates of renal functional preservation. Although patients selected for MW ablation presented with lower baseline eGFR and higher number of comorbidities, postoperative renal decline at 6-months closely mirrored PN and was substantially less than after RN. In multivariable models, RN remained independently associated with greater long-term eGFR loss, whereas MW ablation and PN produced similar renal preservation after adjustment for baseline renal function, hypertension, and diabetes. Baseline eGFR itself was a strong determinant of subsequent decline. This is consistent with recent reviews of MW ablation that report substantially smaller mean decline in eGFR compared to PN (−4.47 vs. −13.09 mL/min/1.73m^2^) [35]. While longer-term renal function outcomes beyond 6-months may be of interest, most studies indicate that the renal functional trajectory stabilizes within the first few months after intervention, and subsequent change is largely driven by non-treatment factors; thus, the 6-month timepoint offers a pragmatic and clinically meaningful comparison of treatment impact [36,37]. These findings underscore that nephron-sparing approaches—whether PN or MW ablation—can maintain renal function in high-risk patients and may mitigate long-term renal and cardiovascular consequences of overtreatment in localized RCC [18,38,39].

Our findings also align with evidence that long-term mortality in SRM frequently is primarily dependent on patient comorbidity and baseline renal function more than RCC, emphasizing the importance of balancing cancer control with physiological reserve and comorbidity burden [7,38,39,40,41]. In this study, comorbidity predicted readmission and renal decline, whereas treatment modality did not meaningfully influence CSS or MFS after adjustment—underscoring the need to weigh oncologic control with physiologic reserve, competing mortality risks, and renal preservation goals rather than focusing solely on maximal local therapy when selecting a treatment modality.

Oncologic outcomes among the three modalities in this study further clarifies the contemporary role of MW ablation. Although MW ablation is not specifically endorsed in current American Urological Association guidelines [42], the National Comprehensive Cancer Network includes MW ablation, as well as other ablation strategies for selected clinical T1a renal masses ≤3 cm [14]. The increasing utilization over the past decade of MW ablation seen in contemporary practice surveys and registry level datasets emphasizes a need for continued oncologic follow-up [43,44]. We found five-year LRFS, MFS, and CSS were excellent across all modalities, with CSS and MFS remaining comparable between MW ablation and PN after multivariable adjustment, paralleling institutional experiences and modern meta-analyses showing comparable CSS outcomes between TA and PN when applied to appropriately selected tumors [6,22,26,28,29]. Local control patterns highlight the trade-off between higher local recurrence risk and the feasibility of effective salvage after MW ablation. LRFS was worse after MW ablation than after PN, yet the majority of recurrences were managed with repeat MW ablation, providing durable secondary local control in all repeat ablation patients. This experience mirrors broader TA literature in which local failure rates are higher than surgery but often salvageable without compromising systemic cancer control. For patients with limited physiologic reserve or marginal renal function, an upfront MW ablation strategy with surveillance and willingness to perform salvage therapy (for less than 5% of patients), offers an acceptable balance between oncologic safety and treatment-related morbidity. Importantly, our series included both T1a and T1b disease, extending prior MW ablation evidence predominantly limited to small (≤4 cm) tumors, as data suggest some T1b lesions can be successfully managed with MW ablation [6]. Although MW ablation demonstrated higher local failure rates, particularly in larger T1b tumors, salvage treatment with repeat ablation resulted in durable local control in all patients who opted for secondary treatment. This increased risk of local recurrence may result in additional imaging, procedures, and patient burden, and the optimal approach to salvage management following local failure remains incompletely defined. Importantly, systemic cancer outcomes (CSS and MFS) remained favorable even among selected T1b patients, highlighting the evolving role of MW ablation especially for older or medically complex patients for whom surgical risk is greater. The overall promising results with MW ablation emphasize a growing consideration for inclusion in guideline-based management as an ablation modality for T1a tumors, while multi-institutional validation and long-term follow-up, especially for T1b tumors is still required.

Interpretation of comparative-effectiveness data in RCC requires recognition of biologic and clinical selection factors. Notably, RN demonstrated worse MFS and CSS likely related to treating a biologically higher-risk population creating a surgical selection bias. Surgically managed patients, particularly those undergoing RN, exhibited higher rates of grade 3–4 tumors (PN 24.5%, RN 40.4%, MW ablation 6.7%) and 7.4% of those patients undergoing PN and 23.8% of RN patients had occult pT3-4 disease, indicating biologic risk enrichment in both surgical cohorts. Although final pathologic stage is unavailable for MW ablation patients, there was likely an underestimation of risk because pathologic stage and margin status are not available to evaluate. In this context, our multivariable findings are notable: nuclear grade was the strongest biological determinant of outcome, with high-grade tumors demonstrating markedly increased risks of recurrence and disease-specific mortality, reaffirming the central prognostic role of nuclear grade in localized RCC. Yet despite this imbalance, CSS and MFS did not appreciably differ between MW ablation and PN. These findings further underscore the importance of pre-treatment renal mass biopsy to better characterize tumor biology, reduce overtreatment of benign disease, and support individualized, risk-adapted management strategies prior to selecting ablation or surgical intervention [45,46]. This parallels findings from modern observational studies suggesting surgery is appropriately favored for anatomically or biologically higher-risk tumors, while TA can achieve comparable systemic cancer control in carefully selected patients [6,28].

This study’s strengths include its large sample size, median follow-up duration of 67 months, inclusion of both T1a and T1b disease, nephrometry scoring, independent radiologic adjudication of local recurrence failures, tissue-confirmed histology in all patients included in oncologic analyses, and direct comparison of MW ablation with both PN and RN. Limitations include retrospective design, lack of pathologic stage and margin status within MW ablation cohort, inherent differences in local recurrence between treatment approaches, residual confounding and treatment selection bias between cohorts creating imbalances between treatment groups despite attempts at adjusting with multivariable modeling and propensity-matched sensitivity analyses. In addition, the relatively low number of oncologic events, particularly for metastasis and cancer-specific death, limits the precision of recurrence risk estimates and underscores the need for feasible prospective, randomized trials to more definitively assess oncologic equivalence between ablation and surgical approaches [47]. Furthermore, these data were obtained at a tertiary center with substantial experience in MW ablation, and outcomes may not be directly generalizable to centers with less experience. Nonetheless, the stability of CSS and MFS, combined with markedly favorable perioperative and renal functional profiles, support MW ablation as a robust nephron-sparing option in contemporary RCC care, particularly for older, comorbid patients or those with limited renal reserve.

## 5. Conclusions

Percutaneous MW ablation offers excellent CSS and MFS, with perioperative and renal functional outcomes that compare favorably with PN in patients with clinical T1 RCC. Although local recurrence was more frequent after MW ablation, particularly among selected T1b tumors, long-term CSS and MFS remained comparable to PN. These findings support consideration of MW ablation as a nephron-sparing treatment option for appropriately selected patients, while recognizing that prospective randomized trials are needed to definitively establish oncologic equivalence and inform guideline-level recommendations.

## Data Availability

Data from this manuscript are available upon reasonable request from the corresponding author.

## Supplementary Materials

The following supporting information can be downloaded at: https://www.mdpi.com/article/10.3390/cancers18020334/s1, Tables S1–S4. Table S1. Baseline Clinical, Demographic, and Tumor Characteristics of patients with pathologically diagnosed RCC (n = 1962). Table S2. Five-Year Local Recurrence–Free Survival by Tumor Size and Treatment Modality. Table S3. Five-Year Metastasis–Free Survival by Tumor Size and Treatment Modality. Table S4. Five-Year Cancer Specific Survival by Tumor Size and Treatment Modality. Table S5. Oncologic Outcomes of the Propensity-Matched Cohorts from 2011–2025. Table S6. Baseline Clinical and Tumor Characteristics of the Propensity-Matched Cohorts. Table S7. Oncologic Outcomes of the Propensity-Matched Cohorts. Figure S1. Flow diagram of inclusion and exclusion criteria by cohort.

## Abbreviations

The following abbreviations are used in this manuscript:

cT1	Clinical T1
RCC	Renal Cell Carcinoma
AS	Active Surveillance
TA	Thermal Ablation
PN	Partial Nephrectomy
RN	Radical Nephrectomy
MW	Microwave
SRM	Small Renal Mass
LOS	Length of Stay
eGFR	Estimated Glomerular Filtration Rate
LRFS	Local Recurrence-Free Survival
MFS	Metastasis-Free Survival
CSS	Cancer-Specific Survival
CCI	Charlson Comorbidity Index
