# Supplementary material for: Percutaneous Microwave Ablation Preserves Renal Function with Similar Long Term Oncologic Outcomes Compared to Surgery for Clinical T1 Renal Cell Carcinoma
Source: Cancers (Basel). 2026 Jan 21;18(2):334. doi: 10.3390/cancers18020334 (PMC12839187; doi:10.3390/cancers18020334)
Supplement: Supplementary file 1 [file cancers-18-00334-s001.zip › cancers-4071331-supplementary.pdf]

Table S1. Baseline Clinical, Demographic, and Tumor Characteristics of patients with pathologically diagnosed RCC (n=1,962)

	MW Ablation N=668	PN N=598	RN N=696	p value MW Ablation vs PN	p value MW Ablation vs RN	p value PN vs RN	p value Overall
**Median Age, y, (IQR)**	67.4 [61.6, 74.1]	55.3 [46.0, 63.0]	61.3 [53.0, 69.1]	<0.001	<0.001	<0.001	<0.001
**Gender: Male n (%)**	464 (69.5%)	373 (62.4%)	456 (65.5%)	0.01	0.13	0.25	0.03
**Race n (%)**							
**White**	611 (91.5%)	557 (93.1%)	640 (92.0%)	0.54	0.66	0.57	0.71
**African American**	31 (4.6%)	22 (3.7%)	26 (3.7%)				
**Other**	26 (3.9%)	19 (3.2%)	30 (4.3%)				
**Median BMI, (IQR)**	31.1 [27.0, 36.8]	30.8 [26.8, 36.2]	30.3 [26.6, 35.1]	0.58	0.01	0.06	0.03
**Smoking History n (%)**	294 (44.0%)	307 (51.3%)	392 (56.3%)	0.01	<0.001	0.08	<0.001
**Diabetes History n (%)**	210 (31.4%)	115 (19.2%)	180 (25.9%)	<0.001	0.03	0.01	<0.001
**Hypertension n (%)**	472 (70.7%)	292 (48.8%)	369 (53.0%)	<0.001	<0.001	0.13	<0.001
**Solitary Kidney n (%)**	56 (8.4%)	14 (2.3%)	6 (0.9%)	<0.001	<0.001	0.04	<0.001
**Bilateral Tumor n (%)**	72 (10.8%)	36 (6.0%)	41 (5.9%)	0.002	0.001	1	<0.001
**Multifocal Tumor n (%)**	45 (6.7%)	36 (6.0%)	43 (6.2%)	0.65	0.74	1	0.86
**Charlson comorbidity index, excluding age, (IQR)**	2 [1, 4]	0 [0, 2]	1 [0, 2]	<0.001	<0.001	<0.001	<0.001
**Median Radiographic tumor diameter, cm, (IQR)**	2.7 [2, 3.5]	2.6 [2, 3.6]	4.6 [3.5, 5.9]	0.934	<0.001	<0.001	<0.001
**Median Nephrometry score (IQR)**	6 [5, 8]	7 [5, 8]	9 [7, 10]	0.001	<0.001	<0.001	<0.001
**Tumor histologic subtype n (%)**							
**Clear cell RCC**	447 (66.9%)	449 (75.2%)	558 (80.2%)	<0.001	<0.001	0.008	<0.001
**Papillary RCC**	131 (19.6%)	87 (14.6%)	78 (11.2%)				
**Chromophobe RCC**	32 (4.8%)	43 (7.2%)	28 (4.0%)				
**RCC Other***	58 (8.7%)	19 (3.2%)	32 (4.6%)				
**Tumor pathologic T stage**							

**pT1a**	N/A	477 (79.8%)	275 (39.5%)	N/A	N/A	<0.001	<0.001
**pT1b**	N/A	73 (12.2%)	238 (34.2%)				
**pT2a**	N/A	4 (0.7%)	15 (2.2%)				
**pT2b**	N/A	0 (0.0%)	2 (0.3%)				
**pT3a**	N/A	41 (6.9%)	163 (23.4%)				
**pT4**	N/A	3 (0.5%)	3 (0.4%)				
**Tumor grade n (%)**							
**1**	76 (13.1%)	52 (9.7%)	77 (12.1%)	<0.001	<0.001	<0.001	<0.001
**2**	423 (73.2%)	345 (64.4%)	319 (50.2%)				
**3**	26 (4.5%)	113 (21.1%)	165 (25.9%)				
**4**	13 (2.2%)	16 (3.0%)	65 (10.2%)				
**Not graded & Unknown**	40 (6.9%)	10 (1.9%)	10 (1.6%)				
**Sarcomatoid features n(%)**	2 (0.3%)	1 (0.2%)	17 (2.4%)	1	<0.001	<0.001	<0.001
**Rhabdoid features n(%)**	5 (0.7%)	3 (0.5%)	25 (3.6%)	0.73	<0.001	<0.001	<0.001
**Surgical approach n (%)**							
**Open**	0 (0.0%)	226 (37.8%)	159 (22.8%)	<0.001	<0.001	<0.001	<0.001
**Laparoscopic/Robotic**	0 (0.0%)	372 (62.3%)	537 (77.2%)				
**Percutaneous**	668 (100.0%)	0 (0.0%)	0 (0.0%)				

MW = Microwave, PN= Partial Nephrectomy, RN = Radical Nephrectomy
* RCC Other Included: Clear Cell Papillary RCC (n=68), RCC Unspecified (n=11), RCC Unclassified (n=19), Collecting Duct Carcinoma (n=2), Renal Medullary Carcinoma (n=1), Translocation RCC (n=5), Succinate dehydrogenase deficient RCC (n=3)

Table S2. Five-Year Local Recurrence–Free Survival by Tumor Size and Treatment Modality

Size category	N (MW Ablation)	5-yr MW Ablation	N (PN)	5-yr PN	N (RN)	5-yr RN	p-value
**0–0.9 cm**	5	100.0% (100.0–100.0)	13	100.0% (100.0–100.0)	3	100.0% (100.0–100.0)	N/A
**1–1.9 cm**	107	97.3% (89.6–99.3)	101	100.0% (100.0–100.0)	13	92.3% (56.6–98.9)	0.07
**2–2.9 cm**	170	98.9% (92.6–99.8)	184	99.2% (94.4–99.9)	73	100.0% (100.0–100.0)	0.82
**3–3.9 cm**	134	97.9% (91.6–99.5)	125	100.0% (100.0–100.0)	108	98.8% (91.7–99.8)	0.41
**4–4.9 cm**	75	87.3% (73.6–94.2)	59	100.0% (100.0–100.0)	133	100.0% (100.0–100.0)	0.0001
**5–5.9 cm**	18	92.3% (56.6–98.9)	23	100.0% (100.0–100.0)	136	100.0% (100.0–100.0)	0.01
**6–7 cm**	11	100.0% (100.0–100.0)	14	100.0% (100.0–100.0)	155	99.3% (94.9–99.9)	0.91

MW = Microwave, PN= Partial Nephrectomy, RN = Radical Nephrectomy

Table S3. Five-Year Metastasis–Free Survival by Tumor Size and Treatment Modality

Size category	N (MW Ablation)	5-yr MW Ablation	N (PN)	5-yr PN	N (RN)	5-yr RN	p-value
**0–0.9 cm**	5	100.0% (100.0–100.0)	13	100.0% (100.0–100.0)	3	100.0% (100.0–100.0)	N/A
**1–1.9 cm**	107	100.0% (100.0–100.0)	101	100.0% (100.0–100.0)	13	92.3% (56.6–98.9)	0.0005
**2–2.9 cm**	170	99.3% (95.2–99.9)	184	99.2% (94.9–99.9)	73	97.5% (83.5–99.6)	0.30
**3–3.9 cm**	134	100.0% (100.0–100.0)	125	100.0% (100.0–100.0)	108	97.7% (91.2–99.4)	0.07
**4–4.9 cm**	75	98.4% (89.4–99.8)	59	100.0% (100.0–100.0)	133	98.3% (93.5–99.6)	0.64
**5–5.9 cm**	18	100.0% (100.0–100.0)	23	100.0% (100.0–100.0)	136	88.8% (80.6–93.6)	0.23
**6–7 cm**	11	100.0% (100.0–100.0)	14	100.0% (100.0–100.0)	155	94.3% (88.3–97.2)	0.53

MW = Microwave, PN= Partial Nephrectomy, RN = Radical Nephrectomy

Table S4. Five-Year Cancer Specific Survival by Tumor Size and Treatment Modality

Size category	N (MW Ablation)	5-yr MW Ablation	N (PN)	5-yr PN	N (RN)	5-yr RN	p-value
**0–0.9 cm**	5	100.0% (100.0–100.0)	13	100.0% (100.0–100.0)	3	100.0% (100.0–100.0)	NA
**1–1.9 cm**	107	99.1% (93.6–99.9)	101	100.0% (100.0–100.0)	13	92.3% (56.6–98.9)	0.03
**2–2.9 cm**	170	99.3% (95.2–99.9)	184	99.2% (94.4–99.9)	73	98.4% (88.9–99.8)	0.71
**3–3.9 cm**	134	100.0% (100.0–100.0)	125	100.0% (100.0–100.0)	108	96.7% (90.2–98.9)	0.02
**4–4.9 cm**	75	98.0% (86.6–99.7)	59	100.0% (100.0–100.0)	133	99.1% (93.9–99.9)	0.65
**5–5.9 cm**	18	100.0% (100.0–100.0)	23	100.0% (100.0–100.0)	136	90.4% (82.8–94.7)	0.25
**6–7 cm**	11	100.0% (100.0–100.0)	14	100.0% (100.0–100.0)	155	87.8% (80.8–92.4)	0.24

MW = Microwave, PN= Partial Nephrectomy, RN = Radical Nephrectomy

Table S5. Oncologic Outcomes of the Propensity-Matched Cohorts from 2011-2025

Comparison		Multivariable	
		HR (95% CI)	p-value
**Local Recurrence Free Survival**			
	**MW Ablation vs PN**	63.31 (7.02 – 571.21)	<0.001
	**RN vs PN**	1.21 (0.10 – 14.22)	0.88
	**Age (years)**	1.02 (0.98 – 1.06)	0.27
	**Tumor size (cm)**	1.31 (1.01 – 1.70)	0.04
	**Histology: Clear Cell Referent:**		
	**Papillary RCC**	0.54 (0.18 – 1.64)	0.28
	**Chromophobe/other RCC**	0.41 (0.10 – 1.76)	0.23
	**High Nuclear Grade (3–4) vs Low (1-2)**	5.79 (2.32 – 14.46)	<0.001
**Metastasis Free Survival**			
	**MW Ablation vs PN**	3.74 (0.84 – 16.56)	0.08
	**RN vs PN**	6.00 (1.70 – 21.18)	0.01
	**Age (years)**	1.03 (1.00 – 1.06)	0.08
	**Tumor size (cm)**	1.40 (1.11 – 1.75)	<0.001
	**Histology: Clear Cell Referent:**		
	**Papillary RCC**	1.03 (0.39 – 2.72)	0.95
	**Chromophobe/other RCC**	0.55 (0.13 – 2.31)	0.42
	**High Nuclear Grade (3–4) vs Low (1-2)**	4.35 (2.06 – 9.19)	<0.001
**Cancer Specific Survival**			
	**MW Ablation vs PN**	1.84 (0.42 – 8.06)	0.42
	**RN vs PN**	3.72 (1.00 – 13.90)	0.05
	**Age (years)**	1.03 (1.00 – 1.07)	0.07
	**Tumor size (cm)**	1.30 (1.00 – 1.70)	0.05
	**Histology: Clear Cell Referent:**		
	**Papillary RCC**	0.45 (0.11 – 1.94)	0.29
	**Chromophobe/other RCC**	1.01 (0.30 – 3.38)	0.98
	**High Nuclear Grade (3–4) vs Low (1-2)**	2.31 (1.05 – 5.09)	0.04

MW = Microwave, PN= Partial Nephrectomy, RN = Radical Nephrectomy.

Table S6. Baseline Clinical and Tumor Characteristics of the Propensity-Matched Cohorts

	Variable	MW Ablation	Surgery	SMD
**MW Ablation vs PN**				
**cT1a (≤4 cm)**				
	Median Age, y, (IQR)	62.8 [58.3–67.3]	61.2 [56.7–66.1]	0.20
	Charlson comorbidity index, excluding age, (IQR)	1 [0–2]	1 [0–2]	0.15
	Preoperatively eGFR	75.6 [61.3–89.9]	74.9 [62.7–87.2]	0.02
	Median Radiographic tumor diameter, cm, (IQR)	2.4 [1.9–3.1]	2.5 [2.0–3.1]	-0.13
	Median Nephrometry score (IQR)	6 [4–7]	6 [4–7]	-0.02
**cT1b (>4–7 cm)**				
	Median Age, y, (IQR)	60.9 [55.7–66.2]	61.6 [53.6–67.5]	0.01
	Charlson comorbidity index, excluding age, (IQR)	1 [0.8–2.0]	0.5 [0–2.0]	0.16
	Preoperatively eGFR	65.4 [52.2–74.9]	69.4 [64.0–80.4]	-0.02
	Median Radiographic tumor diameter, cm, (IQR)	4.5 [4.4–4.9]	4.8 [4.4–5.1]	-0.18
	Median Nephrometry score (IQR)	7.5 [5.8–8.3]	7 [6.0–8.3]	0.00
**MW Ablation vs RN**				
**cT1a (≤4 cm)**				
	Median Age, y, (IQR)	65.0 [59.3–70.1]	63.8 [56.2–69.6]	0.19
	Charlson comorbidity index, excluding age, (IQR)	1 [0–3]	1 [0–2]	0.19
	Preoperatively eGFR	71.5 [59.8–84.6]	71.7 [56.3–83.9]	0.11
	Median Radiographic tumor diameter, cm, (IQR)	2.8 [2.2–3.3]	3.0 [2.4–3.3]	-0.19
	Median Nephrometry score (IQR)	7 [6–8]	8 [6–8]	-0.15
**cT1b (>4–7 cm)**				
	Median Age, y, (IQR)	70.6 [66.2–76.0]	69.1 [65.2–75.5]	0.17
	Charlson comorbidity index, excluding age, (IQR)	2 [1–3]	2 [1–3]	0.17
	Preoperatively eGFR	68.9 [54.2–74.5]	67.1 [57.7–73.7]	-0.02
	Median Radiographic tumor diameter, cm, (IQR)	4.6 [4.4–5.4]	5.0 [4.7–5.4]	-0.19
	Median Nephrometry score (IQR)	8 [6–9]	8 [6–9]	-0.17

MW = Microwave, PN= Partial Nephrectomy, RN = Radical Nephrectomy, SMD = Standardized Mean Difference
Propensity matching was performed within clinical T stage strata using 1:1 matching with restrictions on age, Charlson comorbidity index, baseline eGFR, tumor size, and nephrometry score. Covariate balance was assessed using standardized mean differences, with values <0.20 considered acceptable balance.

Table S7. Oncologic Outcomes of the Propensity-Matched Cohorts

	Variable	Matched N	Events (MW Ablation)	Events (Surgery)	HR (95% CI)	p-value
**MW Ablation vs PN**						
	**cT1a (≤4 cm)**					
	Local Recurrence Free Survival	344	4	1	22.96 (1.95 – 270.69)	0.01
	Metastasis Free Survival	344	1	2	1.71 (0.13 – 22.21)	0.68
	Cancer Specific Survival	344	1	5	0.60 (0.06 – 5.64)	0.66
	**cT1b (>4–7 cm)**					
	Local Recurrence Free Survival	24	0	0	—	—
	Metastasis Free Survival	24	0	1	—	—
	Cancer Specific Survival	24	0	1	—	—
**MW Ablation vs RN**						
	**cT1a (≤4 cm)**					
	Local Recurrence Free Survival	86	2	0	—	—
	Metastasis Free Survival	86	0	1	—	—
	Cancer Specific Survival	86	1	1	2.01 (0.13 – 32.31)	0.62
	**cT1b (>4–7 cm)**					
	Local Recurrence Free Survival	86	2	0	—	—
	Metastasis Free Survival	86	0	6	—	—
	Cancer Specific Survival	86	0	4	—	—

MW = Microwave, PN= Partial Nephrectomy, RN = Radical Nephrectomy
Propensity matching was performed within clinical T stage strata using 1:1 matching with restrictions on age, Charlson comorbidity index, baseline eGFR, tumor size, and nephrometry score.
Categories with no events or events occurring exclusively in one treatment arm were considered not applicable.

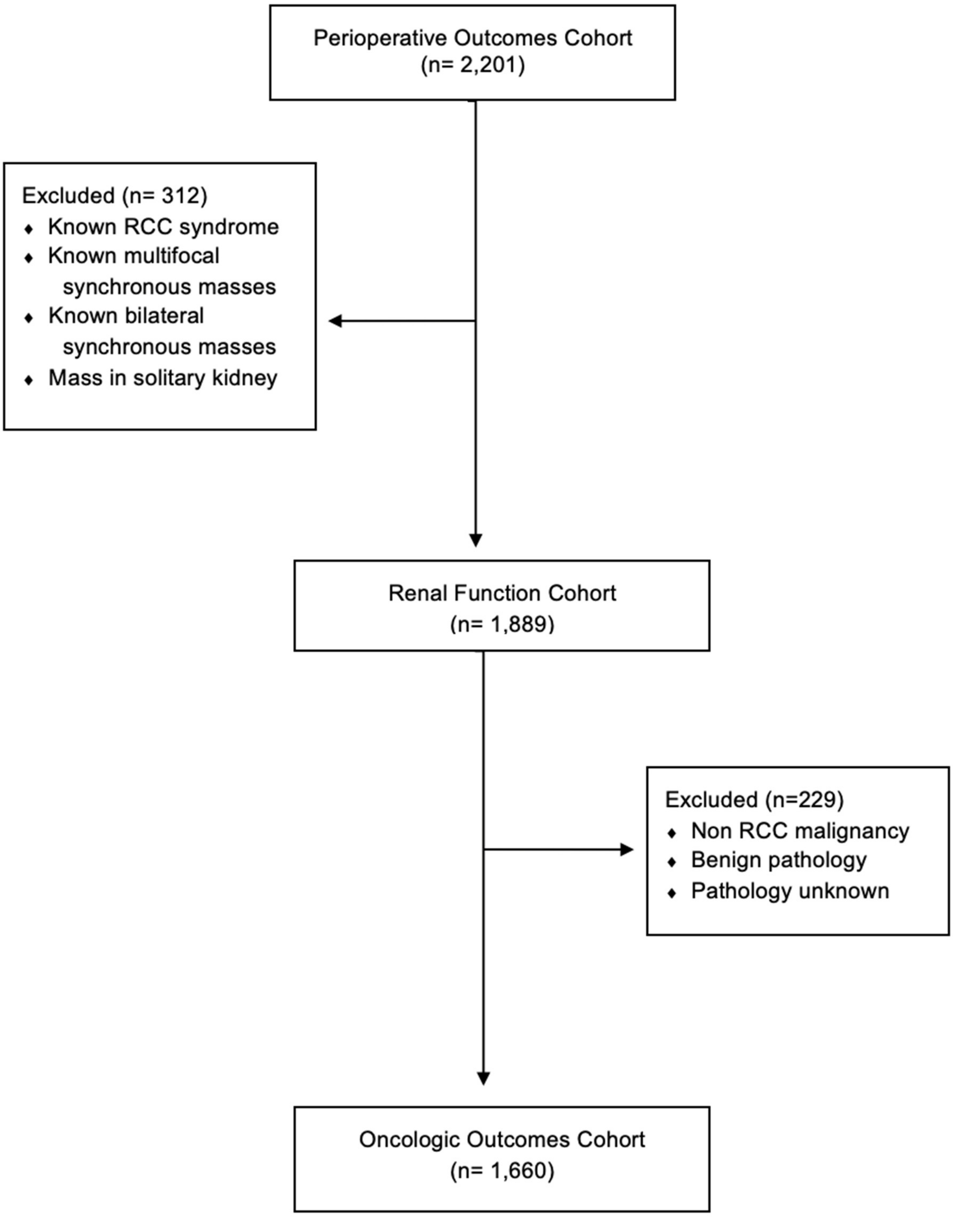

Figure S1.Flow diagram of inclusion and exclusion criteria by cohort.
